# Clinical interpretation of body language and behavioral modifications to recognize pain in domestic mammals

**DOI:** 10.3389/fvets.2025.1679966

**Published:** 2025-10-15

**Authors:** Daniel Mota-Rojas, Alexandra L. Whittaker, Lydia Lanzoni, Cécile Bienboire-Frosini, Adriana Domínguez-Oliva, Alfonso Chay-Canul, Vivian Fischer, Ismael Hernández-Avalos, Andrea Bragaglio, Eleonora Nannoni, Adriana Olmos-Hernández, Arthur Fernandes Bettencourt, Patricia Mora-Medina, Julio Martínez-Burnes, Alejandro Casas-Alvarado, Temple Grandin

**Affiliations:** ^1^Neurophysiology, Behavior and Animal Welfare Assessment, DPAA, Universidad Autónoma Metropolitana (UAM), Mexico City, Mexico; ^2^School of Animal and Veterinary Sciences, Roseworthy Campus, University of Adelaide, Roseworthy, SA, Australia; ^3^Animal Production and Health Division, Food and Agriculture Organization (FAO), Rome, Italy; ^4^EPLFPA-Avignon, Avignon, France; ^5^División Académica de Ciencias Agropecuarias, Universidad Juárez Autónoma de Tabasco, Villahermosa, Mexico; ^6^Department of Animal Science, Federal University of Rio Grande do Sul, Porto Alegre, Brazil; ^7^Facultad de Estudios Superiores Cuautitlán, FESC, Universidad Nacional Autónoma de México (UNAM), Cuautitlán Izcalli, Mexico; ^8^CREA Research Centre for Engineering and Agro-Food Processing, Consiglio per la Ricerca in Agricoltura el’Analisi dell’Economia Agraria, Treviglio, Italy; ^9^Exo Research Organization, Potenza, Italy; ^10^Department of Veterinary Medical Sciences, DIMEVET, University of Bologna, Bologna, Italy; ^11^Division of Biotechnology-Bioterio and Experimental Surgery, Instituto Nacional de Rehabilitación Luis Guillermo Ibarra Ibarra (INR-LGII), Mexico City, Mexico; ^12^Department of Animal Science, Federal University of Santa Maria, Santa Maria, Brazil; ^13^Instituto de Ecología Aplicada, Facultad de Medicina Veterinaria y Zootecnia, Universidad Autónoma de Tamaulipas, Victoria, Mexico; ^14^Department of Animal Science, Colorado State University, Fort Collins, CO, United States

**Keywords:** back arching, ear flattening, tucked tail, companion animals, farm animals

## Abstract

Nonhuman animals use nonverbal cues to communicate their mental state about positive and negative events, including pain. Pain is a multidimensional process that elicits behavioral changes aimed at preventing further damage and promoting healing. These changes include restrictions on movement and/or activity, as well as adopting body postures to relieve pain. Additionally, changes in the ear and tail position have been associated with pain perception and are considered a sign of pain in several domestic species. Thus, this review aims to critically analyze and discuss the behavioral modifications and body language expressions associated with pain in domestic animals, with a particular emphasis on changes in tail position, ear posture, and overall postural dynamics. This review also aims to highlight the essential role of veterinarians and animal scientists in recognizing these subtle non-verbal indicators during clinical evaluation, thereby fostering early detection and effective pain management through more precise observational assessment.

## 1 Introduction

Pain assessment in veterinary medicine requires a multimodal approach that considers parameters beyond physiological and endocrine biomarkers due to its subjective and multidimensional nature (1–4). Some animals, such as horses and rodents, conceal signs of pain due to their prey nature, which forces them not to appear vulnerable to other individuals (5–9). Moreover, non-human animals cannot self-report the presence or intensity of pain (1). Thus, considering the animal’s nonverbal communication cues are essential to accurately evaluate pain (10). Nonverbal communication includes behavioral changes and modifications in body language (8, 11). Behavior refers to the movements and actions performed to respond to stimuli (e.g., withdrawal response, guarding the affected area, or vocalizing) (12). On the other hand, body language refers to changes in the animal’s body posture, as well as limb movements, gestures, and facial expressions (13, 14). Changes in behavior and body language are species-specific and have been recorded in animals exposed to noxious stimuli (15–17).

**Figure 1:**
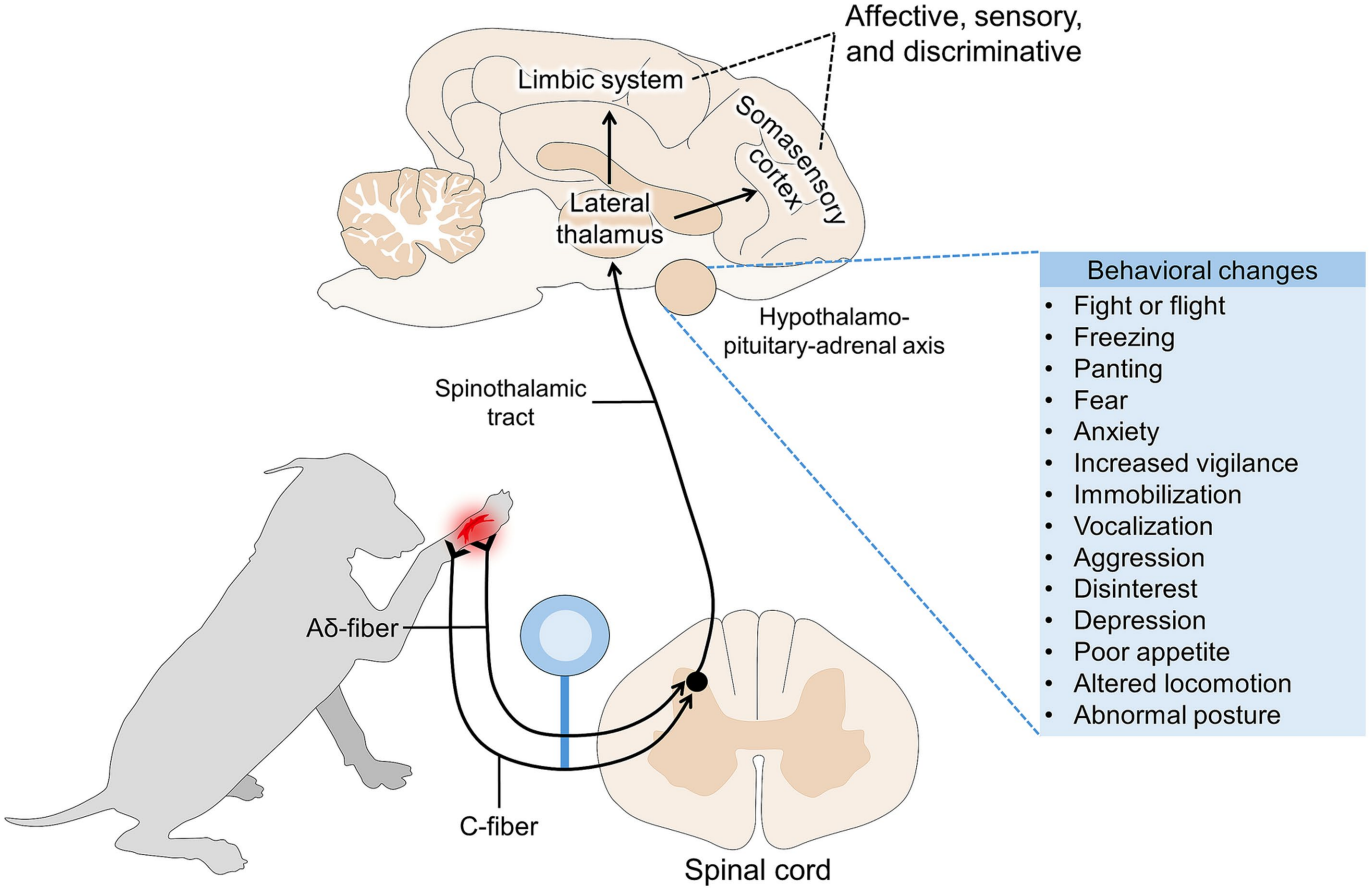
Pain pathway and its association with behavioral responses.

According to the neurobiology of pain, the activation of peripheral nociceptors (nerve fibers specialized in detecting noxious stimuli) and their projection to the brain results in the conscious perception of pain by the somatosensory cortex (Figure 1), also known as the affective component of pain (18–21). Pain demands attentiveness from animals. In consequence, this triggers several active or passive, defensive or reactive behavioral and body posture changes to prevent further damage and promote recovery (6, 22, 23). Due to the neurobiological association between pain processing and both behavioral and body posture changes, these aspects have been integrated into pain assessment scales for domestic mammals, which categorize pain by its intensity and duration (24–26). Additionally, characterization of pain requires consideration of the medical condition (e.g., surgical, traumatic, pathological, physiological) and the anatomical region (e.g., lumbar, abdominal, limbs) to objectively associate certain behaviors with pain (27).

Regardless of the differences between species, in animals such as dogs, cats, horses, pigs, cattle, sheep, and goats, the modification of the position of the ears or tail is considered one of the main changes in body language related to the perception of pain (28–30). However, due to the variability in the expression of pain-associated responses in domestic mammals, assessment using pain scales requires training in the specific behavioral repertoire to detect alterations (31, 32). The complexity of recognizing behaviors and postures associated with pain in animals highlights the role that veterinarians have in promptly detecting pain and educating owners to detect it at home (33).

**Figure 2:**
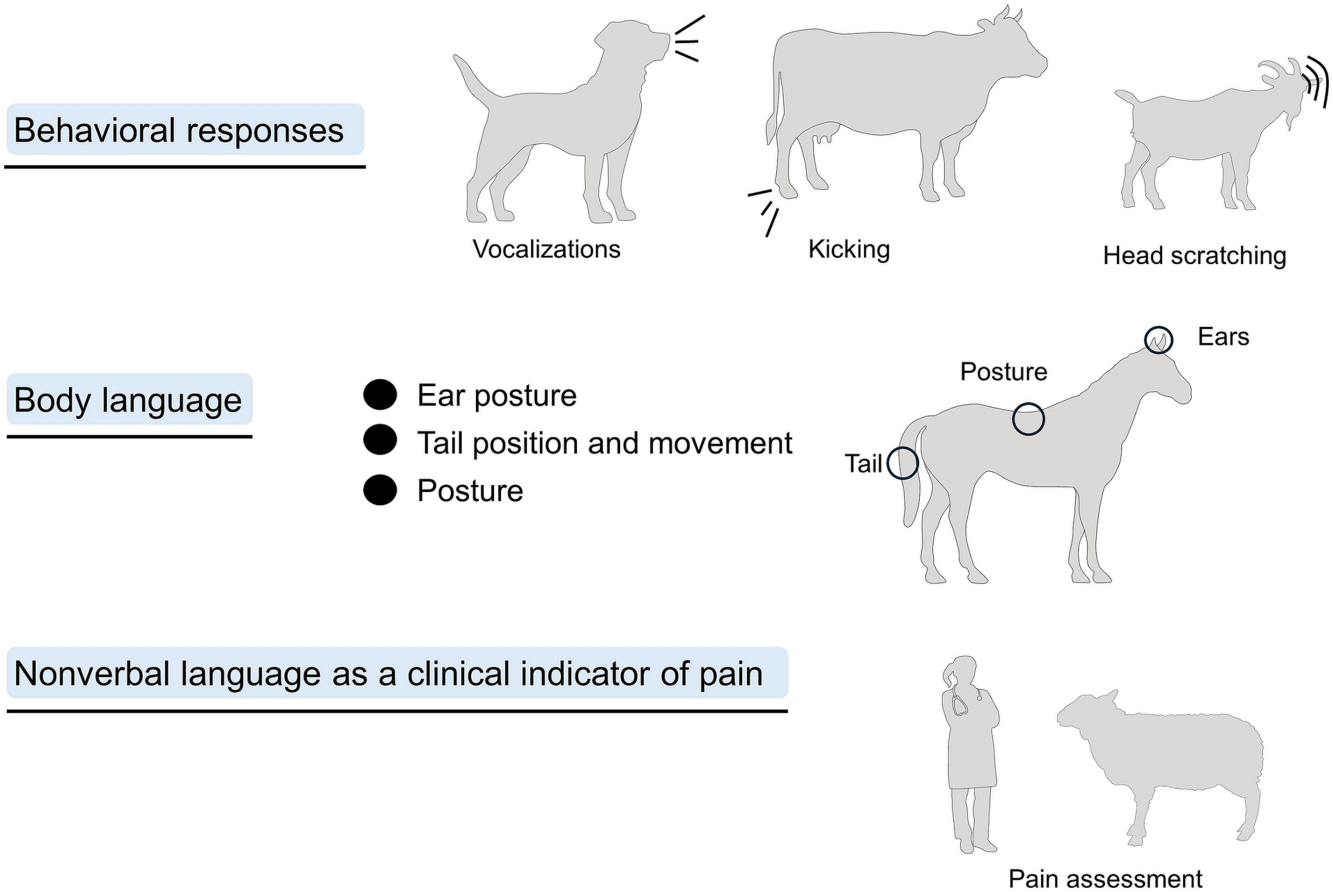
Overall structure of the review, where behavioral responses and body language will be discussed as methods to assess pain.

Through the recognition of the anatomical regions involved in pain processing and how pain manifests as changes in posture and behavior, a clinical and non-invasive evaluation of pain can be obtained. Thus, this review aims to critically analyze and discuss the behavioral modifications and body language expressions associated with pain in domestic animals, with particular emphasis on changes in tail position, ear posture, and overall postural dynamics. This review also aims to underscore the essential role of the veterinarian in recognizing these subtle non-verbal indicators during clinical evaluation, thereby fostering early detection and effective pain management through more precise observational assessment. Moreover, this review also provides practical information that veterinary practitioners can use to assess pain in different domestic mammals. Figure 2 schematizes the overall structure of the review.

## 2 Behavioral responses associated with pain

**Figure 3:**
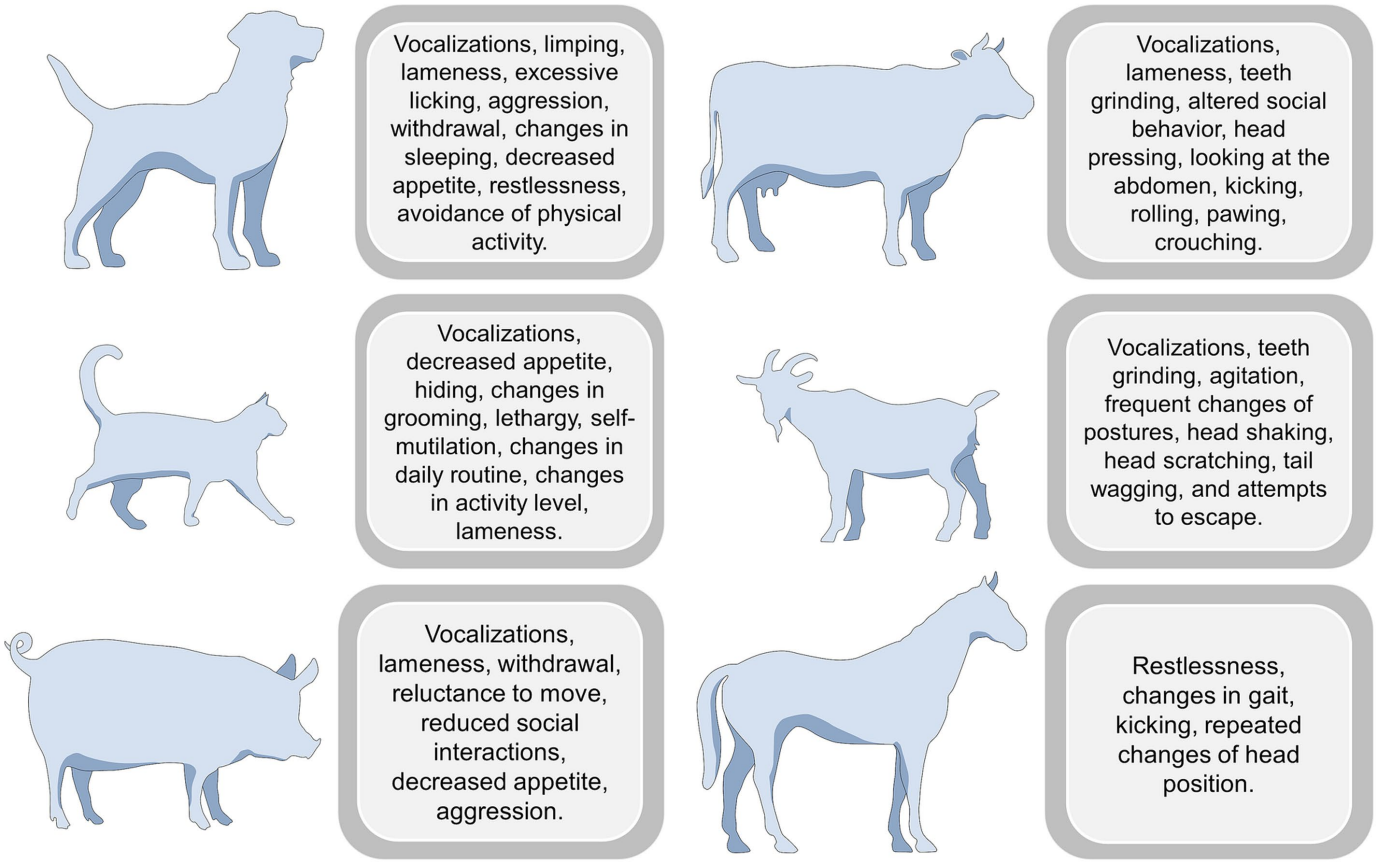
Behavioral responses to pain in different species.

Evaluating the behavioral responses of animals when experiencing pain is considered one of the main noninvasive methods to assess its affective component (34). As previously mentioned, pain-related behaviors in domestic mammals comprise a wide range of activities to reduce the discomfort caused by pain, such as protecting the injured area (35). As mentioned by Camps et al. (36), both losing the presentation of normal behaviors and developing abnormal ones are considered signs of pain. Among the main reported signs in animals experiencing pain are reluctance to move, depression, sleep disturbances, loss of appetite, restlessness, frequent vocalization, licking, biting, scratching, self-mutilation, anxiety, irritability, and aggressiveness (Figure 3) (25, 26, 37–43).

The behavioral response and the change intensity depend on the species and the painful condition. For example, behavioral changes in domestic species such as dogs and cats are the basis for evaluating acute pain (as observed in their respective pain assessment scales) (25, 44). Firth and Haldane (45) were among the first researchers to highlight the importance of pain-related behaviors in dogs by developing a behavior-based scale to assess pain. In this scale, restlessness, vocalization, and reluctance to rise or sit are present in animals with severe surgical pain after ovariohysterectomy (OVH) or castration. Similarly, in Reid et al.’s (44) study, groaning or screaming, growling, and snapping in response to touch, as well as anxiety, fearfulness, or non-responsiveness to stimulation, are considered signs of severe pain during the postsurgical period. These results align with studies reporting that, after OVH and castrations, the response to palpation, reduced movement, and increased frequency of vocalizations are signs of pain regardless of the analgesic treatment (46).

When dogs perceive musculoskeletal pain, particularly in the joints (hip, stifle) or fore/hindlimbs, which represent very common (29–71%) sources of pain (47), main behavioral modifications are reduced general activity and resistance/stiffness to walking (48, 49). These changes may be accompanied by pain-related aggression, as observed in dogs (66.7%) with hip dysplasia (36). Stevens et al. (50) mention that scales scoring appendicular joint pain (in mani, carpi, elbows, shoulders, pes, tarsi, stifles, hips) consider aggression or intention to bite when trying to manipulate the injured area a sign of severe pain.

**Figure 4:**
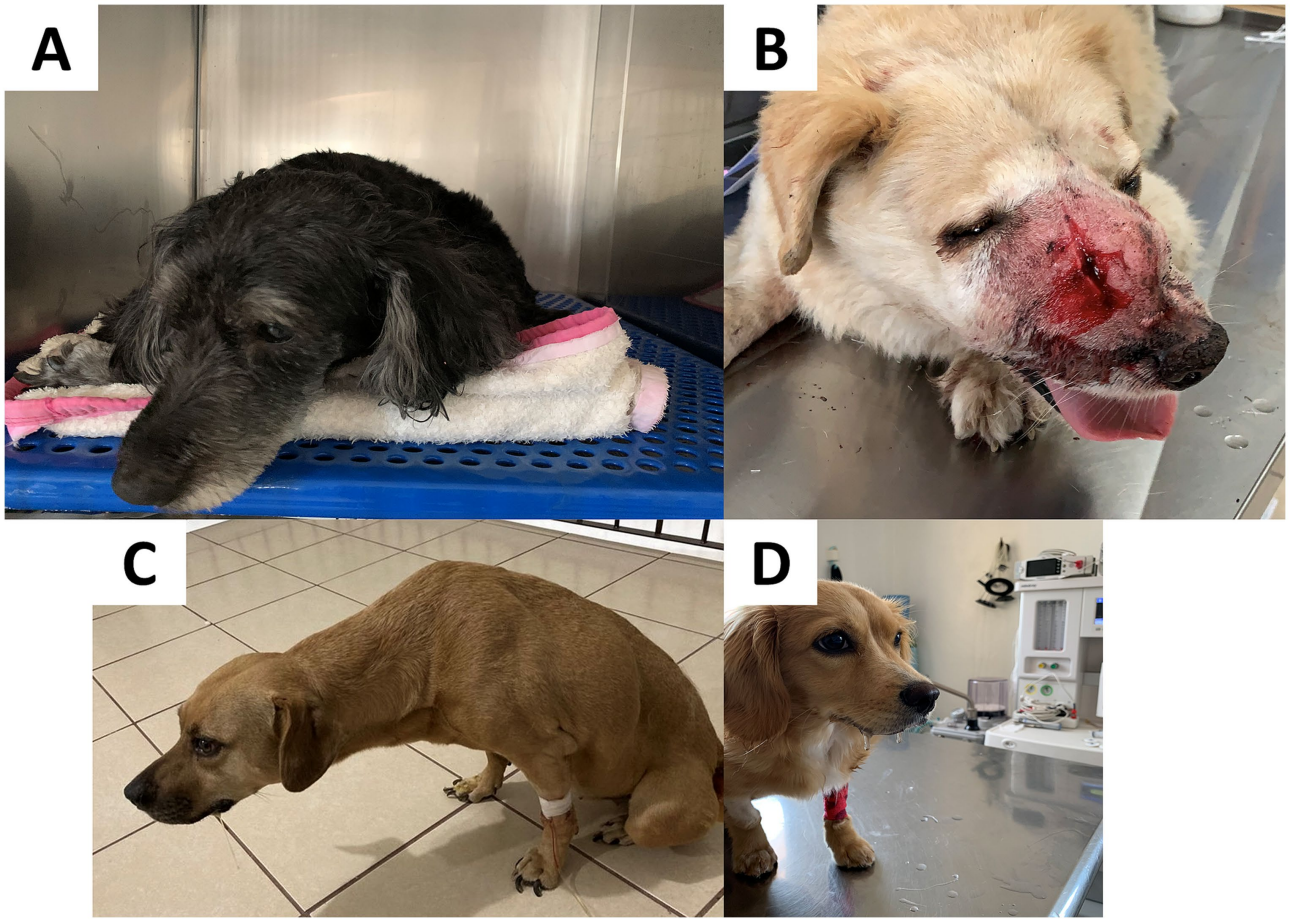
Behavioral changes observed in dogs during hospitalization. **(A)** A patient recovering from pancreatitis. This pathology is associated with restlessness and increased difficulty in adopting a comfortable position to rest. Slower reflexes, body stiffness, changes in appetite, and vocalization can also be observed if the pain is severe. **(B)** A dog diagnosed with a nasal transmissible venereal tumor. This clinical presentation is associated with nasal discharge, sneezing, nosebleeds, respiratory difficulty, and nasal deformity, which can lead to postural changes in patients due to perceived pain. **(C)** A patient with gastroenteritis. Among the behavioral alterations, lethargy, apathy, difficulty in standing, and walking are frequently observed. In addition, dogs may refuse abdominal palpation. Postural changes may include back arching and an orthopneic neck position. **(D)** A patient with excessive salivation is observed after elective OVH. Dogs experiencing postoperative pain may show rapid or abdominal breathing, reluctance to move, abnormal postures when sitting or lying down (e.g., hunched posture with a tense abdomen), and decreased appetite. Photos taken by the authors in a private clinic.

Additionally, this type of pain is also related to unwillingness to learn or participate in training sessions, house-soiling issues, and clinginess to the owner (47). An example is Dodd et al.’s (51) study focusing on military working dogs with lumbosacral stenosis. Twenty-one dogs (32.8%) presented behavioral alterations such as unwillingness or reluctance to jump (38%), self-mutilation in the affected area (25%), anxiety (25%), anorexia (25%), and reluctance to sit (25%). Figure 4 illustrates some examples of behavioral changes in companion dogs and how these can change according to the etiology of pain (e.g., pancreatitis, nasal transmissible venereal tumor, gastroenteritis, and postsurgical pain) (47, 52–56).

In dogs, gastrointestinal pain is associated with compulsive-type behaviors such as star gazing, excessive licking of surfaces, and pica (47). Bécuwe-Bonnet et al. (57) observed copious licking of surfaces (floors, walls, carpets, and furniture) in 59% of dogs diagnosed with eosinophilic and/or lymphoplasmacytic infiltration in the gastrointestinal tract, reduced gastric emptying, irritable bowel syndrome, pancreatitis, and giardiasis. Excessive licking might also progress to self-mutilation in cases of acral dermatitis (58). The overall reduction in activity and mobility observed in animals experiencing all types of pain is related to the protective nature of pain, i.e., its function to prevent further damage, avoid activities that might delay healing, and decrease the inflammatory response that frequently escalates to hyperactivation of peripheral receptors, sensitization, and chronic pain (23).

In the case of cats, contrary to dogs, pain evaluation and recognition of pain-related behaviors are challenging due to their tendency to hide any sign of discomfort unless severe (59). Due to this aspect, the behavioral modifications observed in dogs might not always be present in cats (or be less evident). For example, Monteiro and Steagall (60) mention that mobility changes are less common in domestic felines due to their species-specific behavioral repertoire and inclination to withdraw and hide when threatened. However, among the main behavioral changes related to abdominal pain are a reaction to palpation, decreased appetite, growling, groaning, and decreased grooming (61).

According to Brondani et al. (62), a cat with severe surgical pain licks/bites the surgical wound, reacts aggressively when touching the wound, vocalizes (growls, howls, hisses), shows restlessness and reluctance to move. Similarly, Marangoni et al. (27) mention descriptors such as the level of exploratory behavior, restlessness, grooming, stretching, attention to the wound, growling/hissing, and no interest in food. In the case of chronic pain, a reduction in the animal’s activity is observed, as well as loss of appetite, a tendency to hide or avoid social interaction, and excessive licking of the affected area, decreasing normal grooming (43, 63).

Likewise, some authors refer to key behaviors that help distinguish between painful and nonpainful cats, as reported in kittens subjected to OVH (64). When comparing kittens receiving opioid-free multimodal analgesia with those that did not receive analgesic drugs, animals in pain showed less interest in their surroundings (5 vs. 0%) and played less (7 vs. 35%). Temperament changes are also often reported in cats (91%), as mentioned by Bennett and Morton (65) in adult animals diagnosed with musculoskeletal pain, with reported avoidance of conspecifics and owners. In a case study, aggression due to fearfulness due to arthritic pain in the thoracolumbar spine was reported in a cat presenting house-soiling issues, posturing, and vocalization (47).

In companion animals, these behavioral changes help veterinarians rate the degree of pain. However, studies have shown that dog owners can identify pain through behavioral alterations. For example, 52.6% of owners reported that behavioral signs were very useful to assess pain, and 48.8% of owners reported that identifying these changes was very useful in determining whether they should consult a veterinarian (66). Similarly, in cats, 90% of owners consider it helpful to resort to behavioral evaluations to determine the animal’s degree of pain, and 86% find it helpful to seek veterinary care (67).

In the case of farm species, several instances might cause pain (30, 68). For example, pathological pain due to mastitis or laminitis in ruminants and horses or surgical pain due to castration in piglets and dehorning or disbudding in ruminants, respectively, are accompanied by behavioral modifications that, as seen with dogs, aim to decrease pain perception and promote recovery (69–71). In the first instance, several studies have reported behavioral alterations due to pathological conditions such as mastitis in cattle (72, 73). In this sense, Medrano-Galarza et al. (74) evaluated lying behavior and reactivity during milking (stepping, lifting, and kicking) and its relation to the inflammatory process of the mammary gland due to the presence of bacteria. The authors reported that animals in pain spent statistically significantly less time lying (707.5 min/24 h) than their healthy counterpart (742.5 min/24 h) and that the frequency of lifts and kicks was higher in cows with mastitis (0.70 and 0.10 per minute, respectively). Moreover, Peters et al. (73) evidenced that cows affected by subclinical and clinical mastitis had a lower thermal threshold (higher sensitivity to thermal stimulus) compared with healthy cows, which is observed as a fast foot-lift response at lower temperatures (e.g., 50.9 °C).

Fogsgaard et al. (75) reported that cows suffering from mastitis spent less time lying during the initial phase of the inflammatory disease (720 min/day), and had a higher frequency of kicking (more than 0.70 kicks/min/milking). In addition, Siivonen et al. (76) found that cows spend less time lying on the side with the inflamed udder (control quarter: 40.94 ± 4.60 min; affected quarter: 33.76 ± 2.32 min) and stepped more after an animal model of induced mastitis (up to 1,413 8.6 steps). Another routine procedure on dairy farms that can cause pain and discomfort is drying off, as the accumulation of milk within the mammary gland increases intramammary pressure. Rajala-Schultz et al. (77) observed that cows subjected to gradual drying-off spent more time lying down compared to those undergoing abrupt cessation. Similarly, Maynou et al. (78) reported that the use of acidogenic boluses reduced milk production, which in turn decreased intramammary pressure (55.0 *vs*. 61.9 kg/m/s^2^) and consequently increased lying time. In particular, lying behavior responses in cows are relevant due to their high motivation to lie down (79). In this sense, veterinarians and stockpeople could use lying time in cattle as a potential behavioral marker of pain, as lower lying times are primarily due to the pain and the inflammation of the udder (79, 80), which could help to promptly identify the painful condition and administer pharmacological treatment when necessary.

Tail docking also induces behavioral alterations, as reported in 21- to 42-day-old dairy heifers, tail banding without epidural anesthesia increased restlessness (up to three changes of posture/15 min) (81). Similarly, in crossbred beef heifers, Kroll et al. (82) compared the behavioral response of docked and undocked animals immediately after the procedure. The authors found significantly more steps (up to 200 counts/h), more rear foot stomping (87.2%), and less lying time (approximately 15 min/h) immediately after tail docking than in the subsequent days. A decreased appetite was reported by Eicher et al. (83) in cows after tail docking, reducing the time spent feeding from 17.8 to 13.3% and increasing the frequency of kicking the ground (4%), due to the adoption of alternative behaviors to scare away flies in docked animals. Thus, caudectomy in cattle could have behavioral repercussions because the tail is part of the body language of the species. Additionally, tail docking of dairy cows is declining and is banned in some countries (84). In other species of ruminants, such as sheep, the method of tail docking highly influences the degree of pain perceived by the animals. In this sense, Grant et al. (85) compared tail docking in lambs by rubber ring and hot iron for 90 min after the procedure. The authors found that tail docking by rubber rings significantly increased the frequency of pain-related behaviors such as vocalization (9.9 ± 3.0 animals), number of times the animal changed their lying posture (62.1 ± 5.2%), tail wagging (14.6 ± 2.6 times), kicking/stomping (8.3 ± 1.3 times), and lick/bite the affected area (4.7 ± 0.6 times).

In farm animals, vocalization and its acoustic characteristics during tail docking or castration are considered indicators of pain (86–88), as mentioned by Cordeiro et al. (89), who evaluated the maximum amplitude, pitch frequency, and intensity of vocalization in piglets undergoing castration and tail docking. After the procedure, the maximum amplitude, pitch frequency, and intensity increased by 0.78 Pa, 159 Hz, and 16.9 dB, respectively (89). Similarly, in piglets after hot tail docking, an increase in the frequency and duration of vocalizations was found along with increases in cortisol and β-endorphin levels (90). Hansson et al. (91) reported that the administration of local anesthesia decreases the number and intensity of vocalizations in castrated piglets.

Another common practice on livestock farms is castration. Among the castration methods commonly applied to farm animals are Burdizzo (B), rubber ring (RR), and surgical castration (S), which are frequently compared to a control group subjected only to scrotal handling (H) (92, 93). According to Melches et al. (93), lambs castrated using B and S exhibited more frequent pain-related behaviors during the procedure compared to those in RR and H groups. Moreover, lambs in the S group showed higher cortisol concentrations and a greater occurrence of abnormal postures on the day of castration, along with reduced feed intake and rumination during the first 6 days post-castration relative to the other groups. Similarly, Molony et al. (92) observed in calves that castration using RR was associated with more severe acute and chronic pain, with behavioral indicators of discomfort persisting for up to 42 days. In contrast, castration using S, B, or the combination of B + RR elicited comparatively lower behavioral and physiological stress responses, particularly during the chronic phase.

A study by Yun et al. (94) found that piglet castration without analgesics increased the observation of standing or sitting inactively (102 ± 25.3 counts) and lower frequencies of tail wagging (0.3 ± 0.1) compared to non-castrated animals. Some other behavioral changes were reported in lambs after castration, and similar to tail docking, the method influences the behavioral response. For example, when comparing castration in lambs by cutting with a knife and rubber rings, Lester et al. (95) concluded that behavioral alterations such as abnormal standing/walking and restlessness were predominantly observed in knife-treated lambs within the first four hours after the procedure. In contrast, Maslowska et al. (96) reported that rubber ring castration increased the frequency of active pain behaviors (observable actions when animals experience pain) (a frequency of 110.5) and 44.8% of lambs were more restless and painful than animals that were only handled. Thus, the variability of pain-related behaviors is closely related to the pain source or the method (i.e., castration method), as mentioned by Canozzi et al. (97).

In the case of goats undergoing elective surgical castration, behavioral modifications such as lying down motionless, standing still, and looking at the affected area were considered by Fonseca et al. (98) to develop the Unesp-Botucatu acute pain scale for goats. Vocalizing and teeth grinding have also been reported in adult goats and goat kids during husbandry practices, including castration, disbudding, and dehorning, and during pathological conditions such as lameness or mastitis (99). During disbudding, Kongara et al. (100) summarized that the main behavioral changes observed in kids were head and body shaking, head scratching, and tail shaking. Similar to these findings, Hempstead et al. (101) compared the frequency of pain-related behaviors in disbudded goat kids with cautery iron with a sham group. The results showed an increased frequency of head shaking (31.2 ± 3.11 vs. 17.5 ± 1.79), head scratching (15.8 ± 5.90 vs. 2.2 ± 1.11), head rubbing (4.2 ± 0.77 vs. 0.8 ± 0.27), and body shaking (6.1 ± 0.36 vs. 8.8 ± 0.49) in disbudded animals, which can be used as signs associated with pain. Recognizing these signs is essential to adopt adequate analgesic protocols. For example, Alvarez et al. (102) evaluated the effect of cornual nerve blocks on goat kids undergoing disbudding. The authors evaluated the total behavioral response (including struggle/attempts to escape, vocalizations, and tail movements). It was found that lidocaine administration did not decrease the mean number of said behaviors (control: 59.6 ± 6.8; lidocaine: 52 ± 6.8), suggesting that pain after disbudding should be complemented with other analgesics, such as non-steroidal drugs or general sedation.

Dehorning in cattle has also been associated with pain-related behaviors such as head-shaking, ear flicking, and increased inactivity (103, 104). This has been reported in Holstein calves (4–8 weeks old) after iron-hot dehorning (104). When compared to a control group without receiving analgesic drugs (ketoprofen), treated calves had a lower frequency of head shaking (0.74 ± 0.25 vs. 6.27 ± 2.57) and ear flicking (0.56 ± 0.17 vs. 11.43 ± 3.07) after dehorning. Similarly, the application of lidocaine reduced the frequency of head moving (2.9 ± 0.6 vs. 5.3 ± 1.5), head shaking (1.3 ± 0.6 vs. 27.4 ± 5.9), tail wagging (1.5 ± 0.5 vs. 3.5 ± 0.5), and rearing (0.4 ± 0.2 vs. 1.9 ± 0.5) when compared to a control group of calves dehorned without analgesic (105). Head shaking, ear flicking, and head scratching were also reported in calves dehorned with two methods: cream and hot iron (103). Additionally, in the same animals, a decrease in lying time was observed in comparison with the pre-dehorning period (from approximately 110 min to 75 min), together with decreased playing behavior (from approximately 180 min to 60 min).

Although the discussed pain-related behavioral responses in domestic mammals can differ according to the species and, particularly, to the pain source, these reactions arise to avoid further injury, increase survival chance, and promote healing (32). They are rapid responses of passive or active defense against pain. Therefore, veterinarians and animal handlers should receive training to recognize subtle changes in behavior, as behavioral modifications are one method of communicating pain in animals and may serve as an indicator of their welfare.

## 3 Body language as a tool to assess pain: anatomical structures related to pain perception

### 3.1 Ear posture

Ear position has been used as an indicator of an animal’s affective state, including pain (106–108). The changes in ear posture are related to the neurobiological processing of emotions eliciting different facial expressions according to the context (40, 109). Ear posture depends on motor control from the primary motor cortex and the subnuclei of the facial nerve (VII) (110–113). Although each nervous region has specific functional delimitations, the excitability of the motor cortex leads to positive feedback from the facial nerve, resulting in the contraction or relaxation of the muscles that control ear movement (20, 40). These changes are controlled by the ventral auricular, dorsal auricular, rostral auricular, and caudal auricular muscles (114, 115). However, changes in ear posture alone should not be considered an indicator of pain and must be considered among the several signs that animals show when perceiving pain.

**Figure 5:**
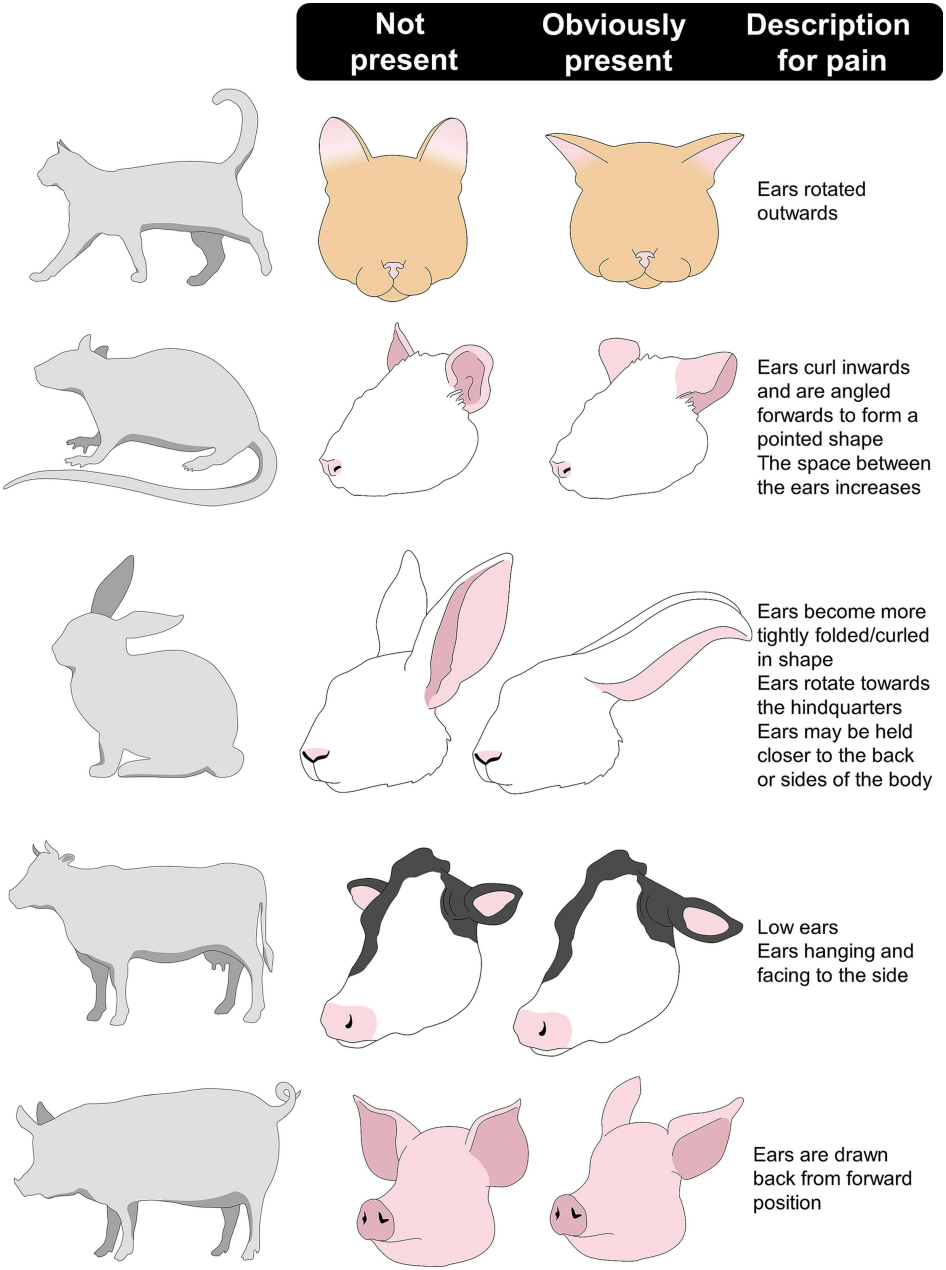
Description of the ear changes in some domestic mammals when perceiving pain.

The importance of ear posture as an indicator of pain in animals is reflected in the current scales that consider it as one of the most noticeable changes when animals are exposed to a noxious stimulus. These scales have been adapted to cats (116), mice (117), rats (114, 118), rabbits (119), pigs (120, 121), sheep (122, 123), goats (124), horses (9), donkeys (125), and cows (126) (Table 1). Although the change and position depend on the species and distinct anatomy, several similarities have been found (125, 127–129). When animals experience pain, stimulation of the auricular muscles causes flattening or retraction of the ears in all species (129–131). For example, a cat with severe pain shows ears that are markedly rotated outwards (132). Rats and mice in pain show ears that are curled, pointed, and/or angled forward or outward (118, 133). For rabbits, ears tightly folded against the neck, pulled back, and flattened are present when the animal is perceiving severe pain (134). Similarly, in farm species such as piglets and goats, severe pain is characterized by the ears drawn back from the forward position and hanging (124, 135, 136), which has also been reported in horses, donkeys, and cows (9) (Figure 5) (109, 118, 132–134, 137, 138).

**Table 1:** Description of ear changes in the currently available Grimace Scales in domestic mammals.

Name	Specie	Ear change	Reference
Calf Grimace Scale	Cattle (*Bos taurus*)	Both ears are backwards, or one ear is directed caudally. The ear pinna cannot be seen, and the angle between the eye commissure, the base of the ear and the tilt of the ears is wider than 90°.	Farghal et al. (221)
Cow Pain Scale	Cattle (*Bos taurus*)	Ears kept straight backwards or very low (“lamb’s ears”).	Gleerup et al. (146)
Donkey Grimace Scale	Donkeys (*Equus asinus*)	Both ears might be back down, one ear forward, and one to the side. One ear to the side and one to the back, or one forward and one down.	Orth et al. (125)
Feline Grimace Scale	Cats (*Felis catus*)	Ears flattened and rotated outwards.	Evangelista et al. (116)
Ferret Grimace Scale	Ferrets (*Mustela putorius furo*)	Ears are pulled back against the body, forming a pointed shape. They may fold over.	Reijgwart et al. (222)
Goat Grimace Scale	Goats (*Capra hircus*)	Ears pinned backwards.	Weeder et al. (124)
Horse Grimace Scale	Horses (*Equus caballus*)	The ears are held stiffly and turned backwards. Thus, the space between the ears may appear wider relative to the baseline.	Dalla Costa et al. (9)
Lamb Grimace Scale	Sheep (*Ovis aries*)	Tense ears pointing backwards or downwards, the inner part of the ear is not visible. Ears appear narrower and dorsally flattened.	Guesgen et al. (123)
Mouse Grimace Scale	Mice (*Mus musculus*)	Ears rotate outwards and/or backwards, away from the face, forming a pointed shape. The space between the ears increases.	Langford et al. (223)
Piglet Grimace Scale	Pigs (*Sus scrofa domesticus*)	Ears drawn back from forward (baseline) position.	Viscardi et al. (135)
Rabbit Grimace Scale	Rabbits (*Oryctolagus cuniculus*)	Ears become more tightly folded/curled in shape. They rotate from facing towards the source of sound to facing towards the hindquarters. Ears may be held closer to the back or sides of the body	Keating et al. (134)
Rat Grimace Scale	Rats (*Rattus norvegicus*)	Ears curl inwards and are angled forward to form a pointed shape and the space between the ears increases.	Sotocinal et al. (118)
Sheep Grimace Scale	Sheep (*Ovis aries*)	Flattened and hanging ears.	Häger et al. (136)
Sow Grimace Scale	Pigs (*Sus scrofa domesticus*)	Ears facing backwards.	Navarro et al. (120)

Clinical examples of pain identification through changes in ear posture and other facial indicators have shown an accuracy of 87% in cats (139). Particularly, as Watanabe et al. (140) mention, ear posture has a good inter-rater reliability score (0.55–0.78) as it is one of the changes caregivers easily observe in cats that underwent procedures such as dental extractions. Holden et al. (141) found that the distance from the midpoint of the two ears is an indicator of pain, where a greater distance between the tips of the ears is considered indicative of pain and correctly classifies between pain-free and painful cats in 95% of cases. Additionally, Merola and Mills (142) mention that in cases of pain due to orthopedic conditions, cancer, urinary tract diseases, or dental issues, flattened ears are frequently observed when perceiving high levels of pain, although it may also be a sign of fear.

In the case of laboratory rodents, Mittal et al. (143) determined the association between pain in sickle mice and changes in the ear position (and other facial indicators). The authors found that exposure to 4 °C caused the ears to move parallel to the neckline, suggesting cold hypersensitivity and pain (scores of up to 1.5). In the same species, evaluations post-vasectomy with and without analgesics found that animals in pain frequently showed ears rotated outwards, and their assessment had an excellent (0.75) reliability score (144). For rabbits, Benato et al. (138) reported that the position and movement of the ears are accurate descriptors of pain. In this sense, flattened ears and lack/diminished ear movement were observed in rabbits after OVH and orchiectomy.

In farm animals, Tallet et al. (145) determined the effect that tail docking has on piglets’ behavior and body posture. In particular, the authors found that immediately after cautery iron docking, piglets held their ears perpendicular to the head-tail axis (70% of animals) and showed more ear posture changes (70%) than non-docked piglets (30 and 20%, respectively). This is similar to what was observed in Danish Holstein dairy cattle during castration, mastitis, or laminitis. In these animals, acute pain was observed as caudal rotation of the ears, along with other changes such as keeping the head below the horizontal axis of the animal, piloerection, arching of the back, and an increased reactivity (146). Additionally, ear posture can also suggest the emotional state of animals, as mentioned by Lambert and Carder (109), who evaluated the ear position of Holstein dairy cows under two different contexts (frustration and excitement). The authors found that cow ears had more changes in ear position during the frustration event (from 14.15 to 16.59 changes/15 min).

In small ruminants such as goats, Weeder et al. (124) analyzed the facial response of goats to induced lameness. Results showed that animals with obvious changes due to pain were characterized by both ears pulled backwards, along with behavioral modifications (e.g., increased lying time). Ear posture changes were also reported by Hussein and Hidayet (147) in goat kids (10–14-day-old) undergoing ear tagging. After the routine procedure, a significant increase in ears backward (from 0.7 ± 0.2 to 11.6 ± 1.7 s), number of posture changes (from 3.3 ± 0.4 to 9.8 ± 0.6), and a decrease in ears plane (e.g., perpendicular to the head-rump axis) (from 25.3 ± 1.5 to 11.8 ± 2.1 s) was observed.

**Figure 6:**
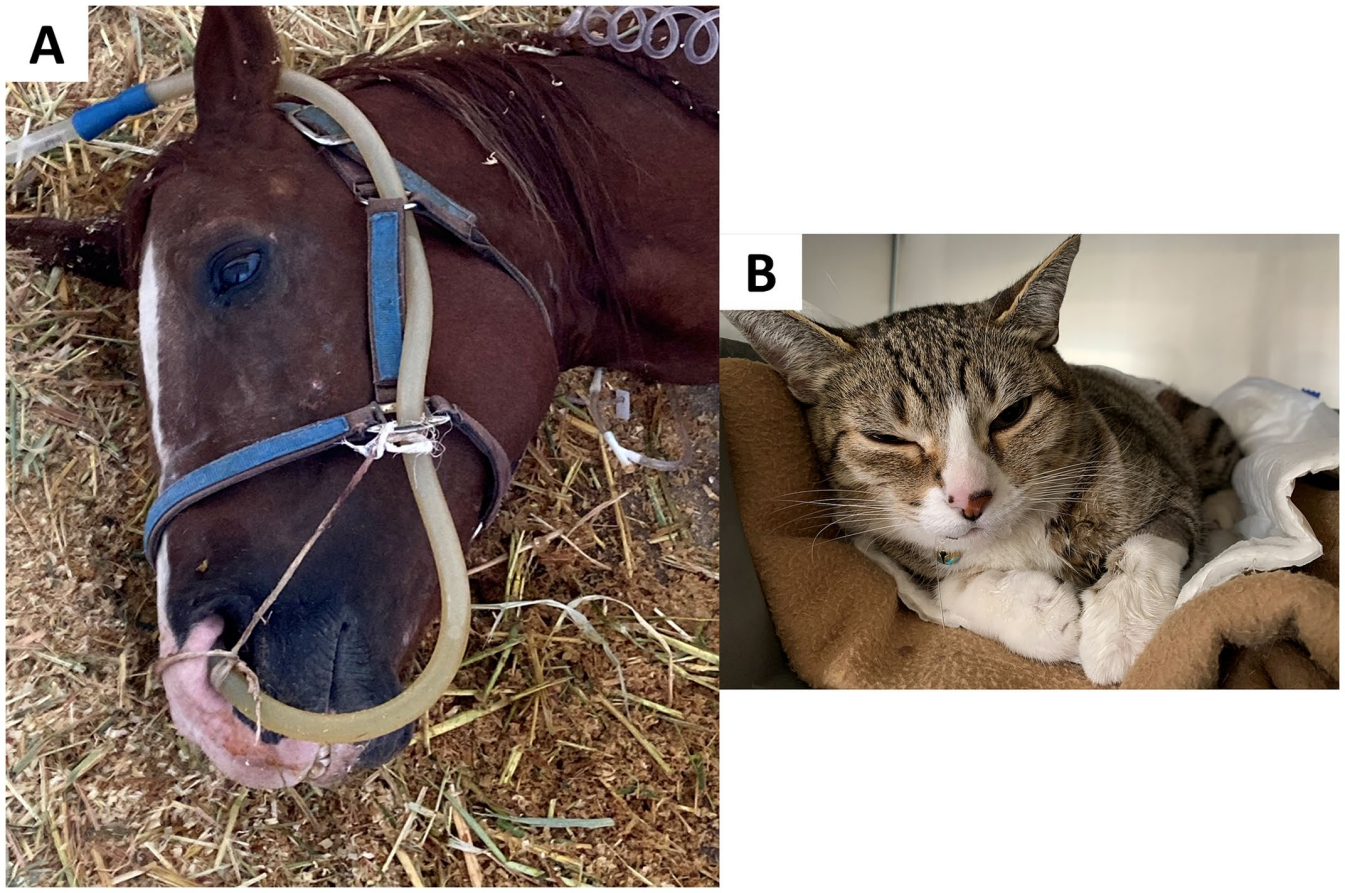
Ear changes in domestic animals during the perception of acute pain. **(A)** A prostrated male Quarter Horse with equine colic due to acute and severe abdominal pain. Facial changes, such as ear flattening, can be observed. **(B)** Feline with idiopathic cystitis. Note the changes in ear position, such as flattening, outward rotation, and being slightly pulled apart. Photos taken by the authors.

Gleerup et al. (148) characterized the changes in ear position of adult horses exposed to experimental acute pain (a tourniquet to the forearm and the topical application of capsaicin). Both stimuli increased the time the horses maintained asymmetrical ears and in a low position (between 54 ± 0.5 and 51 ± 23%), which coincided with the significant increase in the pain assessment scale score. Similarly, Ask et al. (149) evaluated changes in ear position in horses that were administered lipopolysaccharide in the tarsal-crural joint to generate acute pain, highlighting ear flattening and lateral rotation. Additionally, in horses undergoing routine castration under general anesthesia, Dalla Costa et al. (144) found that stiffly backwards ears are associated with pain, with an excellent reliability coefficient of 0.96. Figure 6 shows the ear changes that can be observed in an equine patient with colic syndrome due to pain (150). This figure also shows the changes in the ear position of a feline patient with idiopathic cystitis (151).

### 3.2 Tail position and movement

Tail position and movement have also been considered indicators of pain in animals (152). They have been particularly studied during routine procedures in farm animals such as surgical castration or tail docking (84, 153). During these events, animals in pain maintain their tail stiff, hide it, or swing it abruptly (153).

Changes in tail position or movement respond to adjacent nociceptors that send information through the pudendal and perineal nerves (154). These nerves reach the dorsal root ganglia of the spinal cord and deploy a neuronal and molecular communication circuit at the brain level. Within the brain, the reception and refinement of this information translates into an immediate pain response, coordinated by the amygdala, hypothalamus, and periaqueductal gray matter. The connections of these structures with the motor cortex cause the motor or reflex responses to noxious stimuli (20, 155). The tension of the tail and hiding it between the hindlimbs is due to the contraction of the coccygeus, sacrocaudalis ventralis, dorsalis, and caudae muscles, which stabilize the spine. As a compensatory response to exceeding the nociceptive threshold, the animal modifies the posture of the spine, including the tail, to achieve postural balance and provide greater support and protection (115).

**Figure 7:**
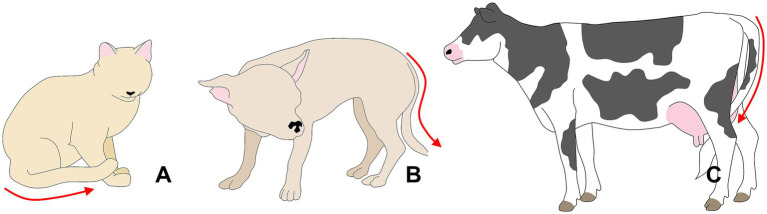
Tail posture as a pain sign. **(A)** In cats, a tail kept or tucked between the hindlimbs, close to the body, is a sign of pain. **(B)** Dogs experiencing pain might exhibit a tucked tail, similar to what is observed in cows **(C)**. In the case of dogs, a tucked tail might also indicate fear. Thus, posture changes need to be interpreted together with other ethological evaluations.

Changes in tail position have been reported in several species (Figure 7) (48, 154, 156, 157). For example, in cats, Pereira et al. (158) mention that tail flicking (along with other behaviors and body postures) indicates pain. For example, in domestic dogs with diseases that generate chronic pain (such as osteoarthritis, cruciate ligament rupture, patellar luxation, pancreatitis, and neuropathic pain) 20% of the owners observed changes in tail posture, keeping it hidden between the pelvic limbs or directed downwards with tension (48). In addition, these changes were accompanied by behaviors associated with pain, such as directed aggression and vocalizations (48). Similarly, the position and laterality of tail movement change depending on the context and can be associated with emotional states (159) such as fear, pain (160), or pleasurable situations (161, 162). In pigs, as reviewed by Camerlink and Ursinus (163), victims of tail biting suffering from pain and (chronic) fear of being targeted keep their tail low and often tucked between their legs.

Miller et al. (164) evaluated tail position in piglets after surgical castration with or without administration of local analgesics. It was found that piglets castrated without local analgesics had a higher frequency of changes in tail position, tail wagging, and maintained a straight (i.e., not curled) tail; in contrast, the non-castrated piglets kept their tail curled and hanging. Similarly, after cautery iron tail docking, piglets maintained an immobile tail in a horizontal position for longer (up to 20 s) than sham-docked piglets (approximately 16 s) (145).

In cattle, vigorous tail swinging vertically or horizontally is suggested as a key indicator for pain recognition; however, a static position or complete immobility of the tail is also associated with pain. In this regard, Tom et al. (165) assessed pain indicators in adult cows undergoing caudectomy with a rubber ring. Cows subjected to this procedure, regardless of analgesic treatment, reduced the frequency of tail shaking up to 6 days after tail-docking (0.8 ± 0.2), in addition to maintaining a straight, ventral position, which results in pressure against the hindquarters (between the anus and vulva) (24 animals). The authors suggested that pressing the tail towards the hindquarters counteracts the painful stimulation caused by the rubber ring and might reduce pain and inflammation (154, 156). Therefore, maintaining the tail static reduces the perception of pain in sensitized tissue.

The importance of tail movements is the reason why production units have focused on the tail to develop sophisticated technologies such as birth sensors (166). These sensors collect the number of times the tail is raised before calving, as this change in body posture is considered an imminent sign of the onset of calving due to the pain promoted by uterine contractions (167). The same posture has been observed in species such as the pig (168) and mice (169). However, tail movements in the peripartum might not always be entirely due to pain.

Therefore, tail position is a key indicator to recognize acute pain, providing valuable information during clinical assessment. However, variability in position and activity is wide across species and animal conditions or affective states. Like ear position, it should be considered an event that manifests together with multiple pain-related signs. Thus, an integrated, multimodal assessment incorporating multiple behavioral and physiological indicators is recommended to increase diagnostic sensitivity and efficacy.

## 4 Assessment of pain through postural changes

**Figure 8:**
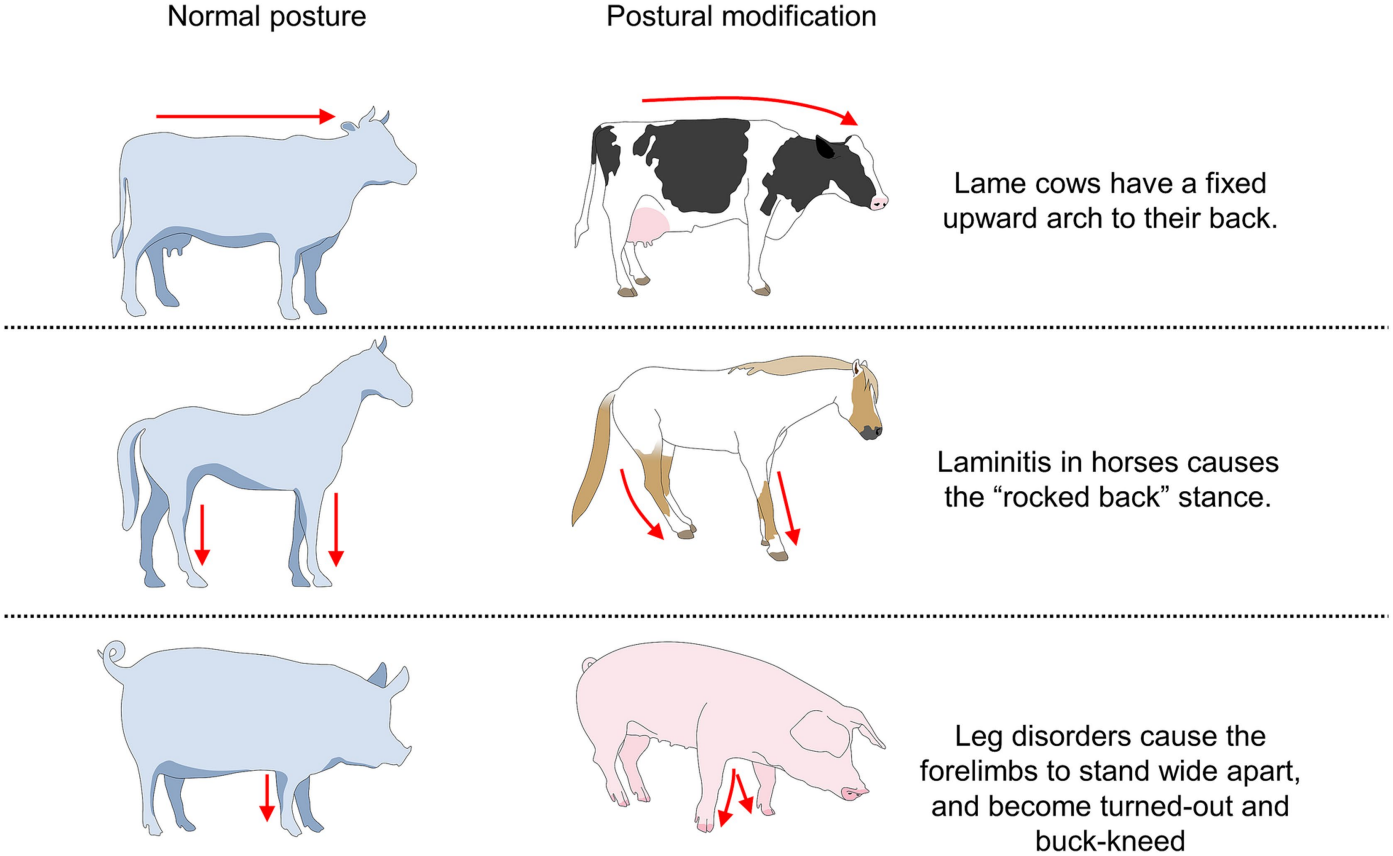
Some examples of body postures associated with pain in domestic animals.

One of the most significant changes in animals experiencing pain is postural alterations to minimize pain perception (30, 170). Back arching, lateral or ventral tilt of the torso, and contraction of the abdominal muscles can indicate the presence of pain (Figure 8). Postural changes are triggered by modifications in the length of muscles, soft tissues, and the musculoskeletal system, influencing spinal alignment to reduce energy expenditure and change body weight distribution to facilitate balance (171, 172). Interception within the musculoskeletal system is carried out by sensory nerves located within the periosteum, spinal cord, and cortical bone (173, 174).

The expression of pain through changes in the position of the trunk or back could be explained by the fact that nerve impulses are projected from peripheral endings processing pain, to the dorsal horn of the spinal cord to the higher structures of the Central Nervous System, specifically the somatosensory cortex (20, 175). Activation of the somatosensory cortex excites relevant regions such as the primary motor cortex, which is involved in the formation of motor actions, such as the withdrawal reflex or postural changes in response to tissue injury (40, 176, 177).

An example of change in posture is during parturition, as observed by Ison et al. (178) periparturient sows show a distinctive posture of back arching, accompanied by hindlimbs pointing forward due to uterine contractions and the expulsion of the piglets. Furthermore, the complete lateral tilt of the torso (animal lying down) was observed for 90% of the time between the onset of uterine contractions and 6 h after the expulsion of the first piglet. The authors state that these behavioral adjustments are indicators of pain and not simply assistive postures in the expulsion of the fetus through the birth canal. This was also observed in periparturient rats by Catheline et al. (169), where the administration of oxytocin, which is a potent intensifier of uterine contractions, increased torso stretching accompanied by abdominal tension (<6 times/min) during parturition. The visceral pain experienced during natural parturition is promoted by several mechanical factors such as uterine contractions, distension, elongation, and tearing of tissue, and pressure applied to adjacent anatomical structures (pelvis and perineum) (179, 180).

**Figure 9:**
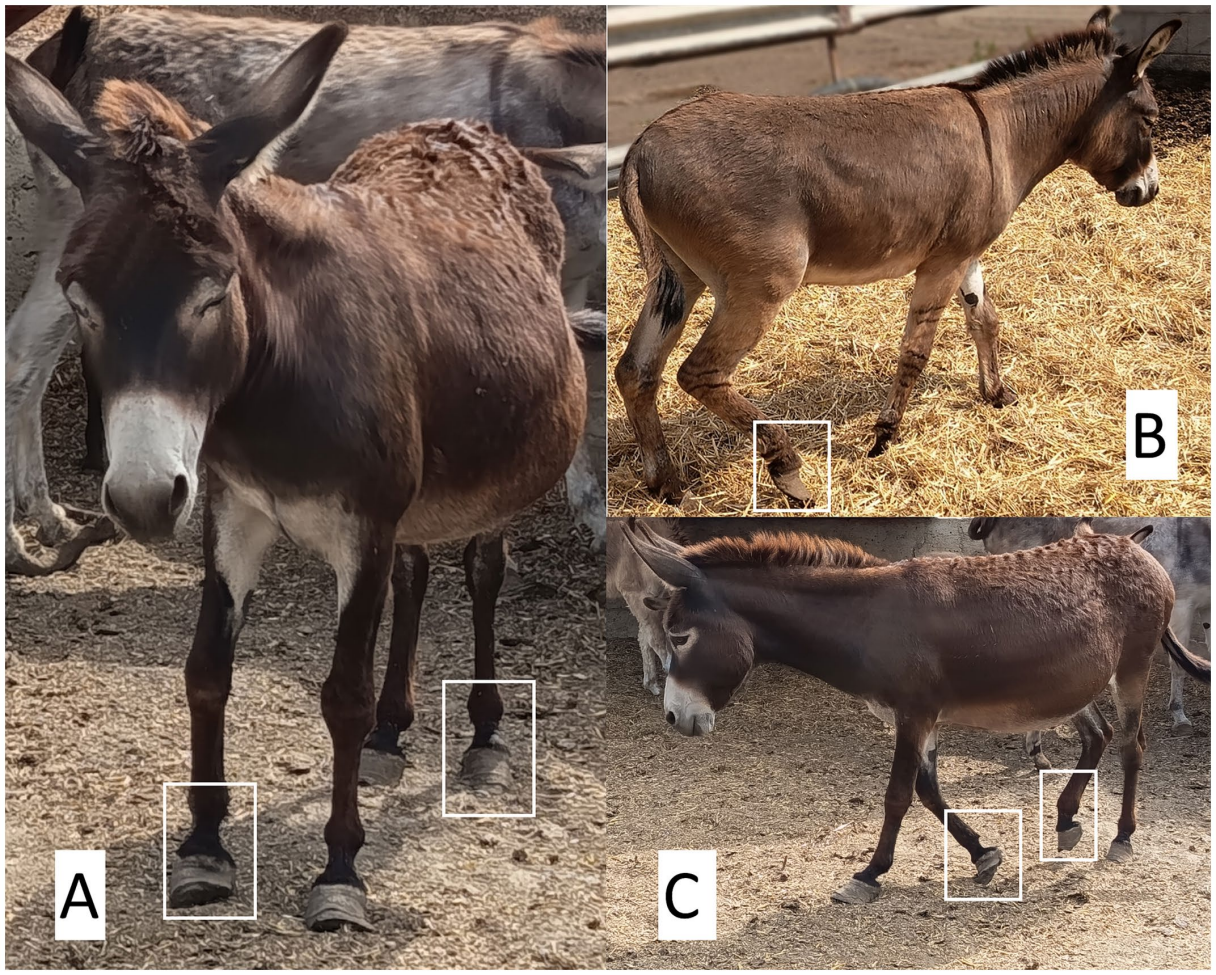
Postural alterations linked to nociception in the metacarpal and metatarsal regions, with clinical signs of laminitis in donkeys. **(A)** The animal displays a posterior shift of body weight, with the forelimbs slightly extended cranially and overextension of the right metacarpal and left metatarsal regions. This abnormal posture results from excessive hoof wall overgrowth. Such a stance is typical of animals experiencing hoof or joint pain, as they attempt to unload the affected areas. A tense facial expression indicative of discomfort is also evident. **(B)** Marked overgrowth of the hoof wall is observed in the right pelvic limb, with clear deformation of the hoof’s natural conformation. This alteration predisposes the animal to chronic pain due to abnormal pressure distribution, joint inflammation, and increased tendinous load. Lack of routine trimming compromises equine biomechanics, significantly altering weight distribution **(C)** The donkey adopts a non-weight-bearing stance, with overextension of the left thoracic limb and complete withdrawal of the right pelvic limb. This postural pattern is consistent with chronic pain, likely associated with laminitis or long-standing podal discomfort. Photos taken by the authors.

The adoption of these postures has been observed in other disorders where visceral pain is intense. For example, in horses with colic syndrome, abdominal pain comes mainly from visceral smooth muscles, which, when undergoing sudden changes such as stretching, tearing, perforation, or strangulation, exceed their nociceptive threshold (181). Fereig (182) associated excessive abdominal stretching with cranial and caudal extension of the fore and hindlimbs, respectively, to relieve mesenteric pressure caused by the accumulation of gas and fluid in the gastrointestinal tract. Laleye et al. (183) mention that early detection of abdominal pain in foals is based primarily on the identification of postural changes or behavioral modifications, among which abnormal body posture of complete lateral tilt and abdominal contraction were frequently reported by owners (47%) and veterinarians (78%). Figure 9 shows an example of postural changes in donkeys suffering from laminitis.

**Figure 10:**
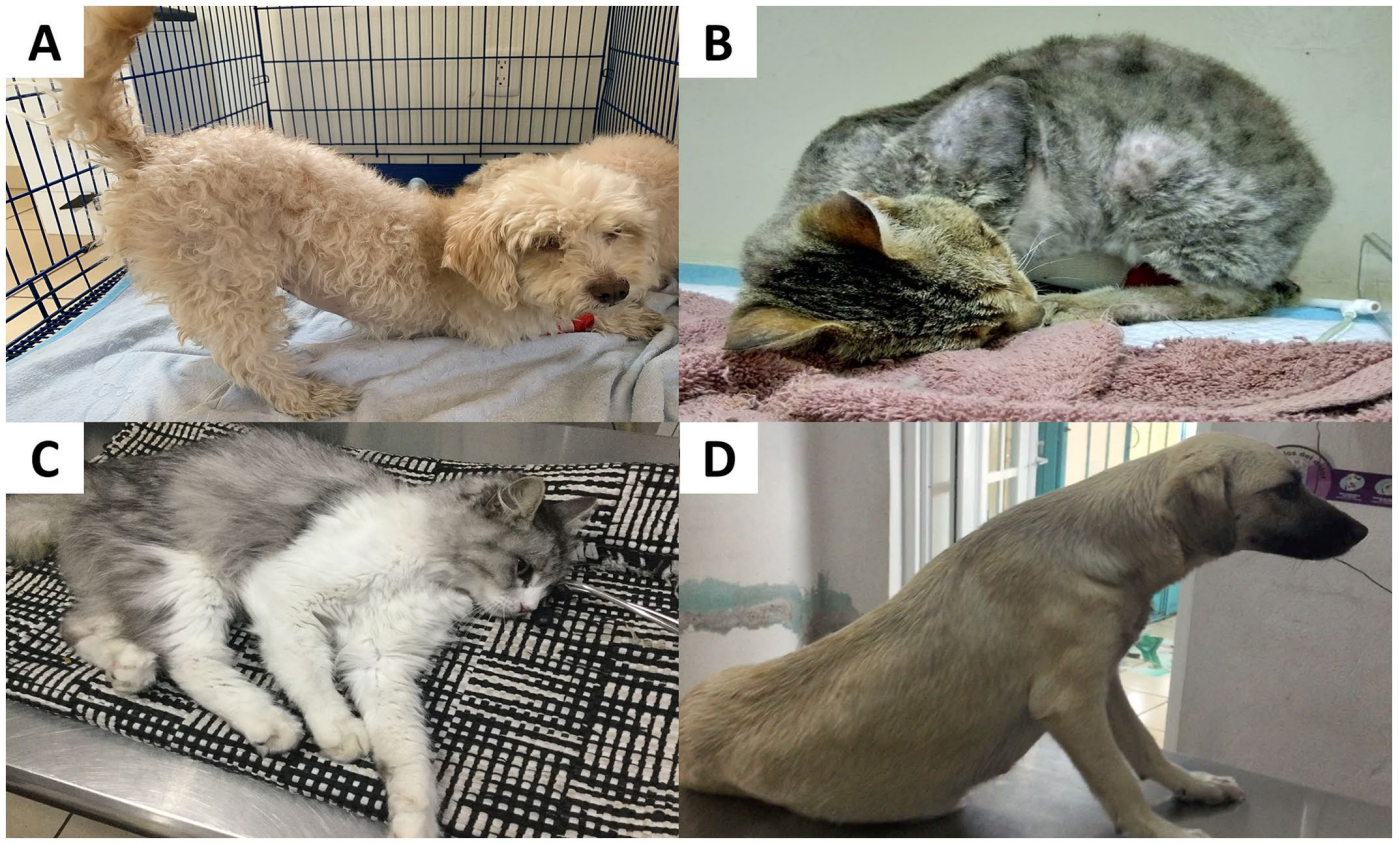
Pain-related postural changes in companion animals. **(A)** Prayer posture in a 2-year-old mixed-breed dog, showing extended pelvic limbs and a lowered head, allowing the pelvis to be raised towards the back. This relieves abdominal pressure when perceiving severe visceral pain. **(B)** Severe acute abdominal pain in a cat. A 6-year-old male cat with severe acute abdominal pain due to chronic kidney disease. The patient maintains a posture with the pelvic and thoracic limbs flexed towards the belly and a lowered head. **(C)** A male cat with the Schiff-Sherrington posture, characterized by extended thoracic limbs and an arched spine (kyphosis), and associated with a spinal injury. **(D)** A 4-year-old dog with a thoracic injury. The dog has an extended neck, with elbows abducted laterally and flexed pelvic limbs. This posture is known as orthopneic stance and occurs in cases of thoracic pain. It is important not to confuse the prayer posture position with the play bow. Play bows occur when a dog is inviting play, whereas the prayer posture is typically associated with discomfort or pain. The dog’s head is usually down when it performs the prayer posture and it is usually up during a play bow. A dog in a playful state is active and energetic, whereas a dog in pain tends to show reduced activity. Photos taken by the authors.

This can also be observed in domestic dogs with the so-called antalgic “prayer posture,” where animals stretch cranially the forelimbs and maintain a convex curvature of the back. By extending the thoracic region, this posture releases the abdominal pressure by the declination of the organs toward the cranial region. This posture is frequently observed when the origin of the pain is visceral and is used as an indicator of postsurgical pain (155). Other postures related to pain in dogs are rigid, hunched or tense, or guarding the affected area (45). Figure 10 shows frequently observed pain-related postures in companion animals due to visceral pain, kidney disease, and spinal and thoracic injury (25, 155, 184–186).

Pain-related postures are not exclusively manifested during visceral pain. For example, mouse models suffering from sickle cell disease adopt an arched back posture in response to a decrease in ambient temperature, a back posture that has also been correlated with other behavioral indicators of pain, such as facial expressions (143). Following abdominal surgical procedures such as OVH in companion animals, marked abdominal contraction is observed, along with exacerbated kyphosis (187), consistent with observations in surgically castrated piglets (164). In particular, the kyphosis manifested during pain in cats caused by musculoskeletal diseases progressively decreases after the administration of analgesic treatment, which suggests that the presentation of antalgic postures is associated with the intensity of pain (65).

**Figure 11:**
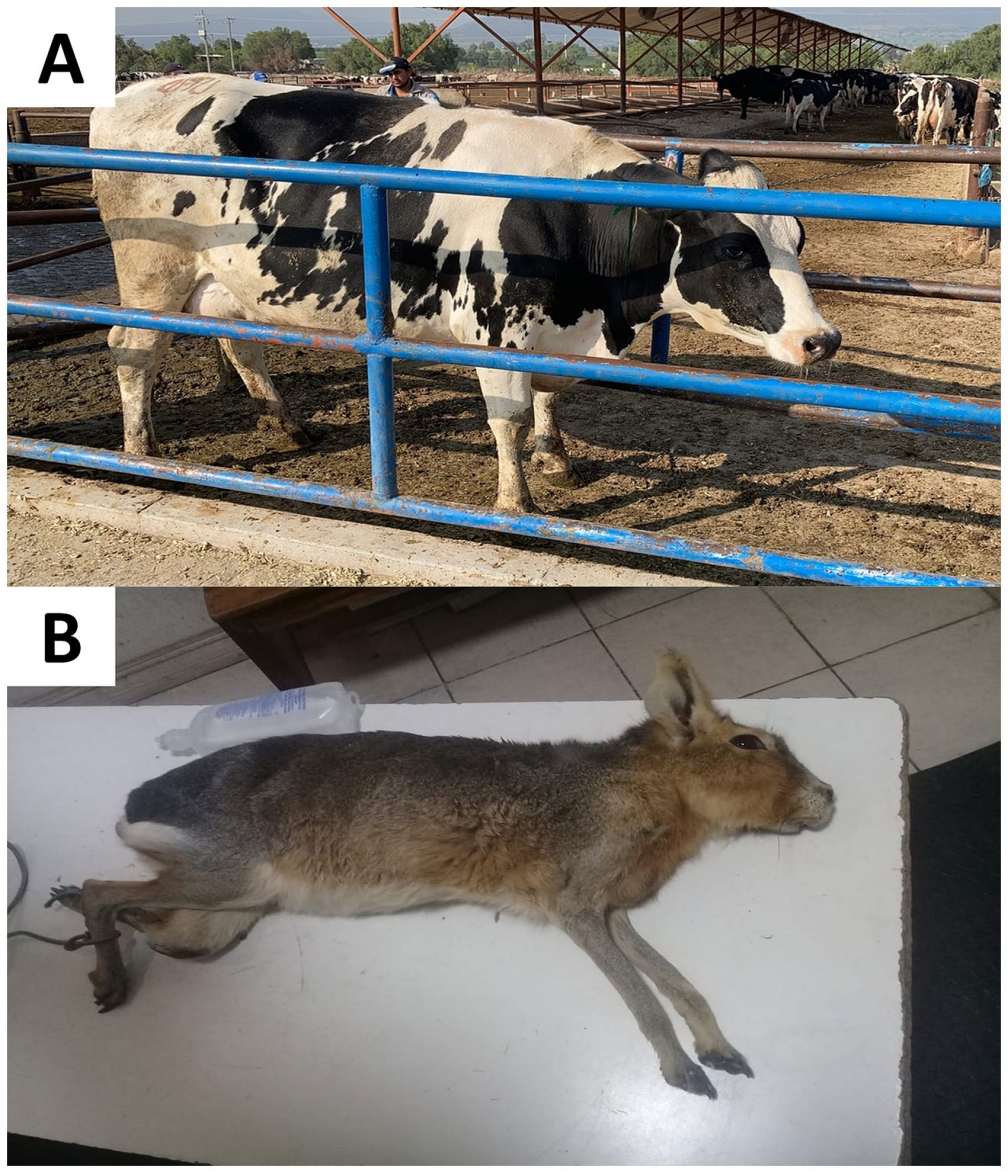
Postural changes in cattle and other species. **(A)** Postural changes in a cow with clinical mastitis. A Holstein dairy cow maintains a low head posture with abducted or clubbed pelvic limbs. This posture helps to reduce contact of the limbs with the udder and diminishes local pain. **(B)** Posture of a rabbit with a fracture in the pelvic limb. A prostration posture and laterally lying on the affected limb apply pressure to the limb to reduce the pain. Photos taken by the authors.

Similarly, abnormal postures occur following routine handling procedures in farm animals. Castration in piglets causes back arching and abdominal tension (164). In small ruminants, Zebaria et al. (188) reported an increase in abnormal standing (standing unsteadily with tail wagging, 6.83%) in kid goats undergoing ear tagging. This is similar to what Fonseca et al. (98) reported in goats subjected to orchiectomy, where the occurrence of an unstable posture increased after the surgery (33.5%). In dairy cows with hoof trauma, Flower and Weary (189) reported marked dorsal arching in animals with plantar hemorrhages and ulcers. These changes were also accompanied by sudden head movements, decreased mobility, and reduced balance in a static state. In another study by Stojkov et al. (190) in cows, dorsal arching was associated with pain caused by inflammation of the uterine wall (metritis), while Rialland et al. (191) associated back arching with gastric problems such as traumatic reticulopericarditis. Figure 11 shows the pain-related postural changes observed in cattle and other species due to mastitis and fractures (192, 193).

Back arching is often accompanied by other body adjustments in the pelvic and thoracic limbs, tail position, neck tension, and head position (30). For example, after surgical castration of bulls, Esteves-Trindade et al. (194) found that the main changes associated with pain were extension of the head and neck, position of the head below the animal’s shoulders, and extended limbs. Recognizing these changes is important for veterinarians and also owners, as reported by Demirtas et al. (48), who evaluated the ability of dog owners to recognize postural changes. These authors observed that, limited joint movement of the caudal vertebrae, arching of the back, and reduced overall activity was present were the most frequently recognized postural changes. Similarly, Laleye et al. (183) evaluated early recognition of colic pain through 66 clinical histories of 40 horses (over 5 years old) and 26 foals (under 4 weeks old). The results indicated that more than 50% of physicians and caregivers use postural modifications as an early sign for colic pain recognition.

## 5 The importance of animals’ nonverbal language as a clinical indicator of pain for veterinarians and animal scientists

Pain recognition and assessment are essential to promote animals’ health and welfare (8, 195, 196). Failure to recognize pain in animals represents a welfare problem due to the physical and mental alterations, including activation of the sympathetic nervous system, immunosuppression, metabolism, and healing processes, as well as increased morbidity, disease progression, and prolonged recovery periods in surgical patients (2, 197). In human medicine, pain assessment is performed through verbal or written communication with the patient (198). In contrast, in veterinary medicine, pain is identified through nonverbal communication, such as changes in physiological and endocrine parameters, body language, and behavior (56).

Pain assessment and management require the veterinarian’s knowledge and objectivity. Therefore, physicians need to incorporate behavioral and postural indicators associated with pain into their daily practice (8, 30). The changes observed in animals are an integrated response aimed at reducing the painful stimulus (199). Moreover, owners need knowledge and awareness of pain behaviors as they are key for the early recognition, assessment, and management of pain (48, 200–202). Therefore, veterinarians need to identify and familiarize themselves with animal behaviors to detect and categorize pain, although factors such as environment, species, age, body condition, and type of disease must be considered (16, 203). Although behavioral scales exist to assess pain, surveys indicate that 73% of veterinarians consider these methods inadequate and have difficulty recognizing behavioral changes (204, 205), which has a direct impact on patients’ quality of life and welfare (206).

Although the study of these behavioral and body posture indicators has been explored in several domestic species, the anatomical differences must be considered to accurately evaluate pain. These changes should not be considered in isolation but as a part of a complementary evaluation considering physiological parameters. Therefore, it would be appropriate to investigate whether including these changes in assessment scales improves the sensitivity of these tools, as has been observed with facial expression (207). Similarly, standardizing changes in ear/tail position and postures for each species and each pain-inducing event is necessary, which could help increase the specificity and sensitivity and obtain an objective pain assessment. The development of multidimensional scales that consider both physiological and behavioral/body posture/facial expression parameters could be the best option to comprehensively evaluate pain in domestic mammals. For example, the Colorado State University Canine and Feline Pain Scale or the University of Melbourne Pain Scale consider physiological, behavioral, and postural responses to acute pain (45, 208, 209).

Although behavioral, postural, and facial recognition of pain can be performed manually by clinicians or stockpeople, automated techniques have been explored for multiple domestic species to increase the accuracy of the evaluation and prevent subjectivity. For example, in companion animals, the Facial Action Coding System for cats (catFACS) was used as an anatomical basis for a machine learning model to recognize pain in cats undergoing ovariohysterectomy (210). The accuracy of the technique was above 72%, indicating its usefulness for automating pain detection. Similar accuracy was reported by Martvel et al. (211), who used artificial intelligence to detect pain in cats by establishing 48 facial landmarks in videos. The authors reported an accuracy of over 70% in recognizing feline acute postsurgical pain. Furthermore, AI and machine learning techniques can help to differentiate breeds and cephalic types in addition to pain (212). Breed-specific morphology highly influences pain recognition in companion animals (213). This is particularly relevant for domestic dogs, as breed-specific face anatomy makes it challenging to recognize pain through facial cues (214). However, Zhu et al. (215) have recently proposed the application of machine learning to automatically identify pain in dogs.

Similarly, the adoption of techniques known as “precision livestock farming” or instruments that use artificial intelligence techniques can help objectively and automatically recognize changes in ear or tail position in farm species. An example is Feighelstein et al. (216), who used deep learning to detect pain from lateral images of horses undergoing routine castration. Using the Horse Grimace Scale (HGS) to embed the Facial Action Units (previously described in Table 1), authors reported an accuracy between 73 and 79% to detect equine pain. This might improve animal management and welfare in routine procedures that are still considered “not as painful.” Similarly, Lencioni et al. (217) developed a machine vision algorithm to detect acute pain in horses after surgical castration. Through facial expression, the authors found an overall accuracy of 75.8% when classifying pain into three categories (not present, moderately present, and obviously present, according to the HGS), or 88.3% when representing absent/present pain. Recent studies have suggested that the use of “regions of interest” instead of “facial landmarks” when using automatic detection of pain could increase the feasibility of adopting artificial intelligence in animal pain detection (218). Moreover, although most of the research is focused on identifying facial changes associated with pain –in horses–, Kil et al. reported a sensitivity of above 80% to detect behavioral changes in horses using machine learning (e.g., analysis of wither, tail, and nose changes).

In ruminants, Salzer et al. (219) developed an automatic warning system to identify mild pain (capsaicin application) in cows. Through a machine-learning algorithm, the authors were able to identify that decreased rumination and restlessness are present in animals experiencing pain with an accuracy of 82%. Additionally, micro expressions have also been adapted to computer vision methods to detect painful conditions such as lameness, metritis, mastitis, and pre-calving pain with an average precision of 83%. In other species, such as goats, Chiavaccini et al. (220) detected acute pain (due to conditions such as castration, mastectomy, dental cleaning, among others) through the analysis of raw facial video footage with machine learning. “Painful” and “non-painful” goats were differentiated with an accuracy of 60%. When automatically analyzing facial expressions of pain in sheep, studies have reported that artificial intelligence outperforms human experts, which has significant applications in farms.

Deep learning-based models and artificial intelligence are still under development for several species (224). However, these methods reduce human bias and the need to manually extract information, which is time-consuming. Therefore, these methods are current alternatives to improve pain assessment in domestic mammals for improving animal welfare, while preserving the importance of training veterinarians and animal caregivers to correctly interpret animal behavior and body language.

## 6 Conclusion

Animal body language serves as a means of understanding the emotional state of animals in response to positive and negative stimuli, such as pain. In domestic animals, variations in behavioral responses such as vocalizations, grooming, scratching, avoidance, escape, tonic immobility, as well as aggression, among other behaviors, are associated with the perception of pain. Additionally, about farm animals, changes in ear and tail position and in the overall posture have been reported to be indicative of pain in animals suffering from pain arising from, e.g., laminitis, visceral involvement, or routine painful procedures. Understanding these signals as a nonverbal communication of pain allows the efficient identification of pain for timely intervention and optimized management.
